# Comparison of Modified Meek Technique with Standard Mesh Method in Patients with Third Degree Burns

**DOI:** 10.29252/wjps.9.3.267

**Published:** 2020-09

**Authors:** Mostafa Dahmardehei, Reza Vaghardoost, Mahdy Saboury, Hamze Zarei, Shahriar Saboury, Mehdi Molaei, Jalal Seyyedi, Abdulbaset Maleknejad

**Affiliations:** 1 Burn Research Center, Iran University of Medical Sciences, Tehran, Iran;; 2 Department of Plastic and Reconstructive Surgery, St. Fatima Hospital, School of Medicine, Iran University of Medical Sciences, Tehran, Iran;; 3 Department of Plastic and Reconstructive Surgery, Imam Ali Hospital, School of Medicine, Zahedan University of Medical Sciences, Zahedan, Iran;; 4 Department of Surgery, Firoozgar Hospital, Iran University of Medical Sciences, Tehran, Iran;; 5 Burn Research Center, Zahedan University of Medical Sciences, Zahedan, Iran

**Keywords:** KEYWORDS

## Abstract

**BACKGROUND:**

Covering burn wounds, especially high surface area burns has been always a challenge for surgeons. The Meek technique has been introduced to increase the covering area. There is paucity of clinical trials comparing the Meek technique and mesh in the same individuals to assess it efficacy.

**METHODS:**

In a case-control study, 20 patients with grade III burns who underwent the Meek technique and mesh in different areas/limbs were enrolled. Expansion rate, re-epithelization, operation time, wound infection, graft failure, etc. were compared between the two groups.

**RESULTS:**

Among patients, 18 were males and 2 were females. The mean of total body surface area (TBSA) was 36.9±16.6%. Mean time of re-epithelialization in the Meek group was 2.8±2.5 months and in the mesh group was 5.0±2.1 months (*p*=0.01). Operation time was shorter in modified Meek technique (*p*=0.04). Expansion ratio was higher in modified Meek technique (*p*=0.04). Local wound infection rates were slightly different without a statistically significant difference.

**CONCLUSION:**

Meek technique provided higher surface area coverage in comparison to mesh; in addition to faster re-epithelization. Therefore, it is recommended to consider the Meek technique as a routine procedure, especially those with high surface area burns.

## INTRODUCTION

Over 300 million people worldwide die each year from burn injuries. In third degree burns (or full thickness of the skin burns), all the layers of the skin including the nerves are involved.^1^^,^^2^ Sepsis frequently occur in deep burns, especially if total burned area is significant. Loss of skin leads to dehydration and infection predisposition. Therefore, wound coverage as soon as possible is very important in burn care. Major electrolyte imbalances occur including hyponatremia, hyperkaliemia, metabolic acidosis and primary renal failure, which could be prevented to a large extent by burn wound coverage.^3^^-^^6^

Dermal grafts are widely used in the treatment of chronic and acute wounds. Mid-thickness or partially thickened grafts comprise part of the dermis and epidermis. Autologous grafts (autografts) are transferred from one body area to another. Allogeneic grafts (allografts and hemografts) are transmitted to host from an unidentified living donor or cadaver, and xenogenic grafts (heterografts) are transferred from one species to another (such as pigs).^7^^-^^9^ Partial-thickness grafts require less blood support to restore skin function. Besides, dermal component of the full thickness grafts provides a good mechanical strength and provides better contraction to the wound, resulting in an increased aesthetics.^10^^,^^11^

In burns with larger Total Body Surface Area (TBSA), lack of adequate donor sites is a great obstacle. In 1958, a technique was introduced to overcome this problem as a skin graft expansion method including small graft island to cover a broader wound area. Later in 1964, meshing of full thickness grafts was introduced. Thereafter in 1993, its use was increased again due to introduction of the modified meek technique.^12^^,^^13^ Meek and mesh methods were later compared. Expansion ratio in Meek was 1 to 9 and in mesh was 1 to 6. It was shown that the Meek technique was appropriate for covering large burn wounds, even despite granulating wounds with poor conditions.^14^

In a large clinical study on 37 patients, the efficacy of Meek technique was assessed over a 5-year period. Burn surface area in this study was as high as 72.9%. The mean number of required surgeries was 1.84 and the survival rate after 5 years was 92%. It was shown that the Meek technique could cover large skin burn wounds with good long term outcomes.^15^ In a systematic review published in 2018 on Meek technique, 24 articles were evaluated. It was concluded that Meek micro-grafting can be used despite a poor wound vascularity with more promising outcomes, probably owing to lower nutritional demand of the graft islands. Hence, it is also more appropriate in case of underlying diseases like diabetes mellitus. It was demonstrated that the Meek can be used in major burns, especially those involving more than 30% TBSA. This technique was suitable in case of inadequate donor site as it provided a higher expansion ratio. Despite the fact, the only disadvantage reported was long-term dotted graft appearance.^16^

Despite the fact, most of these studies performed on Meek micro-grafting were cross-sectional studies and there was paucity of clinical trials comparing Meek and mesh techniques, especially at different body areas of the same individuals. Performing the two techniques concurrently in the same individuals removed some bias regarding host factors and allowed for a more conclusive assessment. Therefore, the aim of this study was to compare expansion rate, re-epithelization, operation time, wound infection, graft failure, etc. between the Meek and mesh techniques at different parts of the same individuals. 

## MATERIALS AND METHODS

In a case-control study, all patients with third degree burns who referred to the referral burn center of St. Fatima Hospital, School of Medicine, Iran University of Medical Sciences, Tehran, Iran entered the study. All patients were first resuscitated and burn wound debridement was performed in either one or repeated sessions in the operation room. Burn wound debridement was performed using the Humby knife removing all necrotic tissue reaching to active punctate bleeding. Hemostasis was ensured using adrenaline-soaked gauzes, dressings and repeated physical and hemodynamic assessments.

Exclusion criteria were smoking, grades I, II, IV burns, those with diabetes or collagen vascular diseases or any apparent wound infection. However, those who were not consent to enter the study were also excluded. This study was approved in the Ethics Committee of Iran University of Medical Sciences (IR.IUMS.FMD.REC.1398.281). All the study steps were performed in accordance with the Helsinki Declaration. All consents were obtained by attending physicians and all patients agreed to the analysis and publication of the data and images. All patients were informed and explained about the burn degree and their treatment plan.

**Fig. 1:**
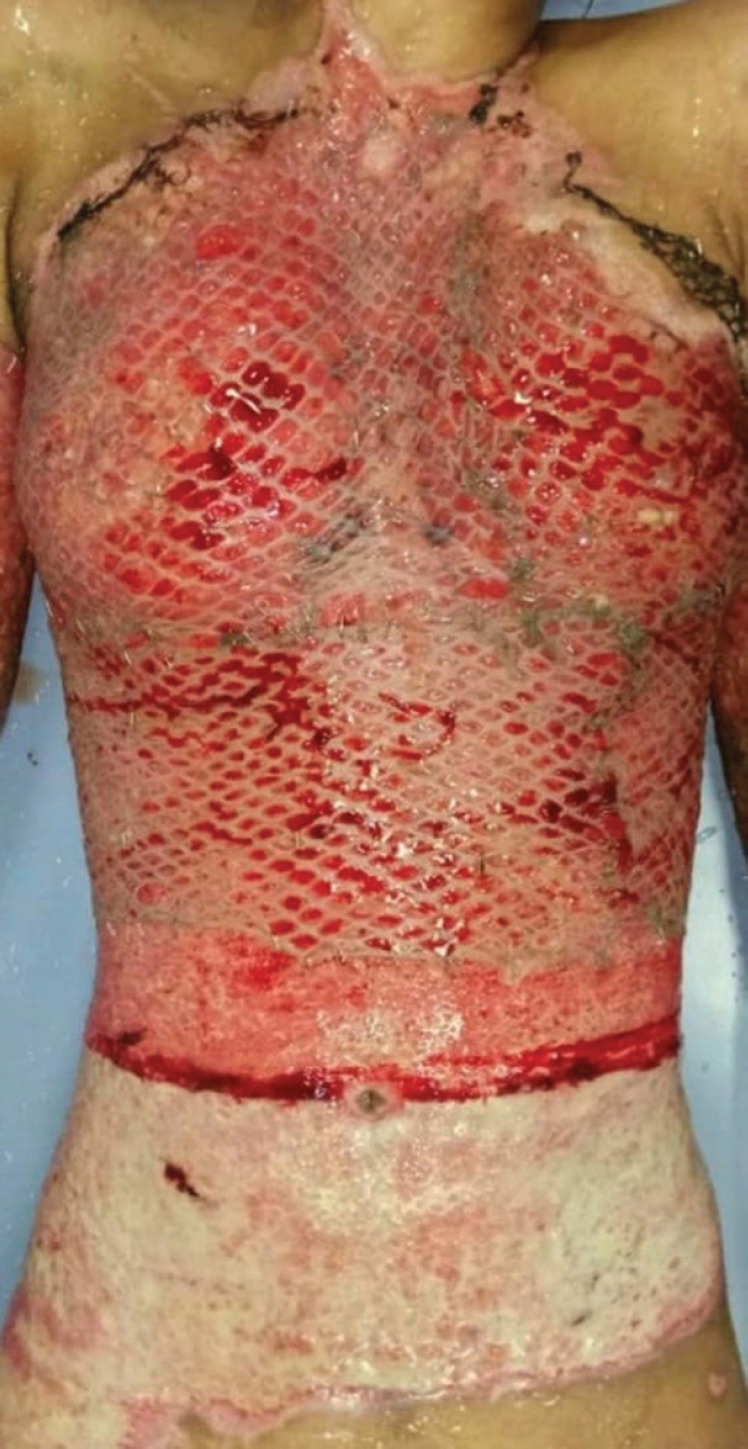
Upper chest covered with meshed skin graft

**Fig. 2:**
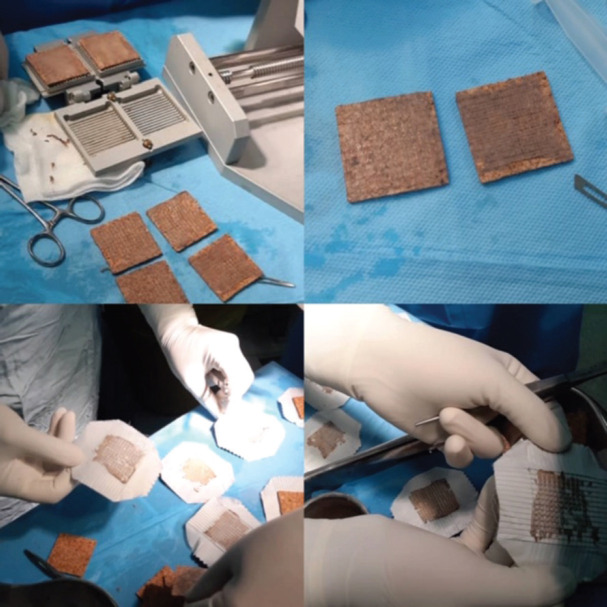
Preparation of skin graft with modified Meek technique

The donor site was removed by a Dermatome blade based on the graft required. Part of the removed graft was meshed (1;4, 1;6) according to the size of the wound. Grafts were placed at wounds of one area and fixed using staplers (Figure 1). In the next step, the second limb/area underwent modified Meek technique. First the grafts were cut using the Meek mesher (Humeca) to yield “postage stamp” squares of 3×3 mm. Expansion rates of 1;4 and 1;6 were used. The islands were fixed using an adhesive spray on the epidermal side. A polyamide pleated sheath with aluminum backing was applied. The plate was removed after 5 days depending on the surgeon examination (Figure 2).

Patients’ data including demographic information (age, gender), TBSA percentage, expansion ratio, hospital stay, graft rejection, contracture, hyperpigmentation, operation times, re-epithelization time and the operation time were recorded. Data entered SPSS software (SPSS Inc. Chicago, IL, USA) and were statistically analyzed using Fisher Exact and Mann-Whitney tests. A p value less than 0.05 was considered statistically significant.

## RESULTS

Eighteen patients (90%) were male and 2 (10%) were female. Mean age of patients was 26.5±5.7 years with a range of 19-54 years. In this study, 20 patients (40 limbs or separate areas) with grade 3 burns who were candidates for skin grafts were included. Demographic data of patients was presented in Table 1. Modified Meek and mesh graft techniques were performed in all patients and the results were compared. 

**Table 1:** Baseline characteristics of the participants

**Variable**	**Value**
Gender (M/F)	18/2 (90%)
Age (Mean±SD)	26.5±5.7
Mechanism of burn	
scalds Flame Chemical	8 (40%) 9 (45%) 3 (15%)
Inhalation Injury	
Yes No	2 (10%) 18 (90%)
TBSA burn	36.9±16.6
Hospital stay, Mean±SD (Days)	45.4±6.8 (21-135)

**Fig. 3:**
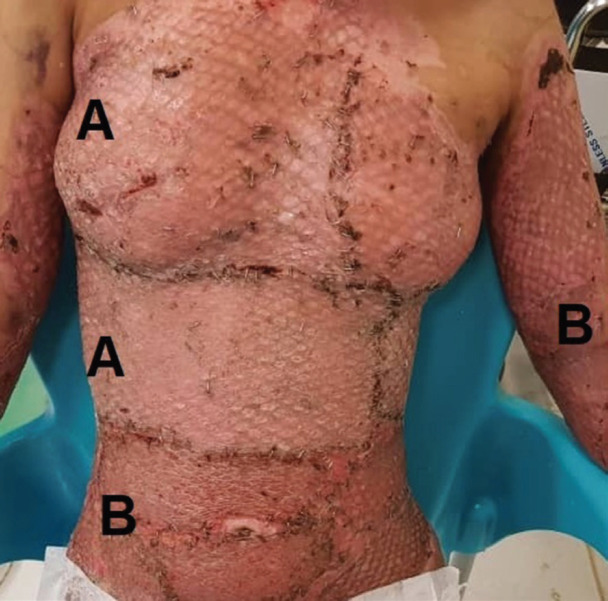
**A:** Mesh grafted areas. **B:** Areas covered with modified Meek technique (18 days post operation).

**Fig. 4:**
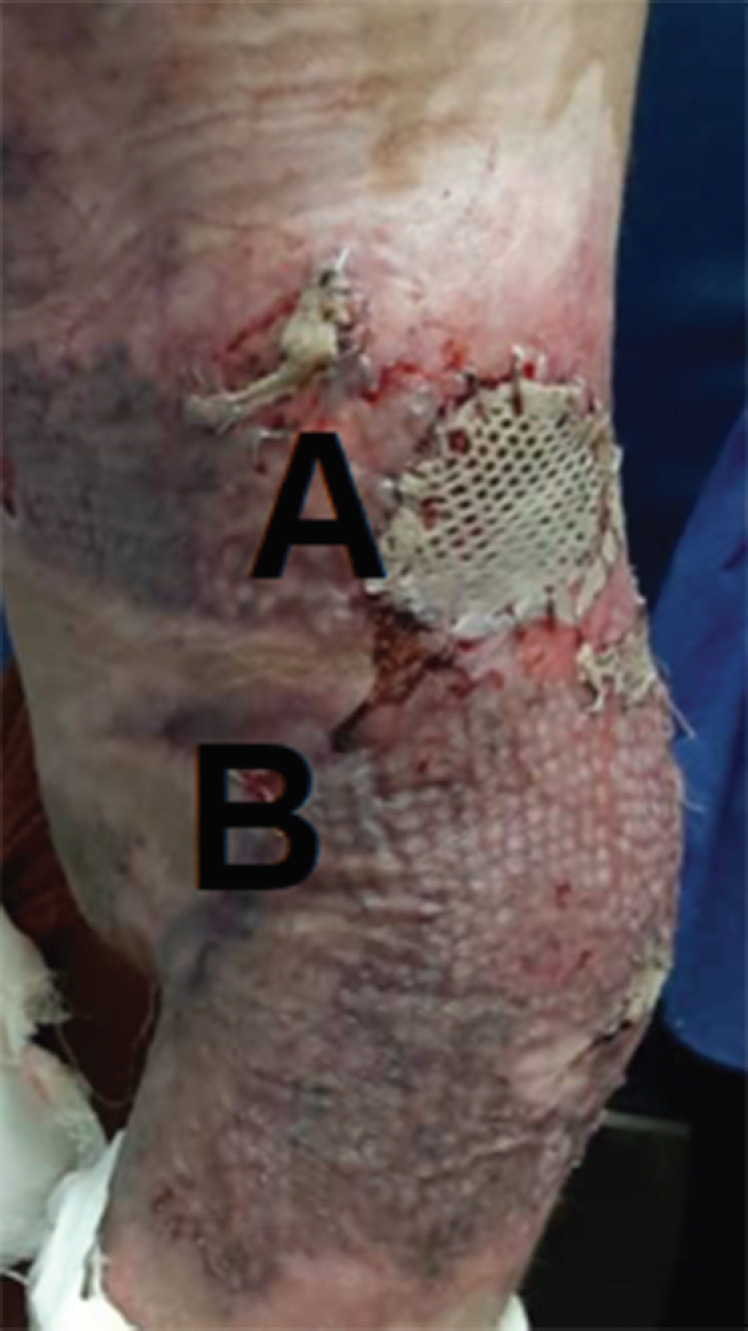
**A:** Mesh grafted areas. **B:** Areas covered with modified Meek technique (27 days post operation).

The mean TBSA was 36.9±16.6% surface area. Mean percentage of body surface area covered by the Meek technique was 39% and for the mesh technique was 30%. The rate of graft rejection in modified Meek graft technique was 3 (15%) and 5 in mesh procedure (25%). The difference was not statistically significant (*p*>0.05). In modified Meek technique, 11 wounds (55%) and in mesh technique, only 3 (15%) were epithelialized in the first month. This difference was statistically significant (*p*=0.03, Figure 3). Mean time of re-epithelialization in the Meek group was 2.8±2.5 months and in the mesh group was 5.0±2.1 months, with a statistically significant difference (*p*=0.01, Figure 4).

The average duration of operation in modified Meek technique was 26.9±5.4 minutes and in mesh technique was 32.1±7.4 minutes. Operation time was shorter in modified Meek technique with a statistically significant difference (*p*=0.036). The mean expansion ratio in modified Meek technique was 5.7±1.9 compared to 4.2±1.8 in the mesh technique. Expansion ratio was higher in modified Meek technique with a statistically significant difference (*p*=0.04). In modified Meek group, 3 cases of local wound infection and in mesh group, 5 cases were reported without a statistically significant difference. A summary of outcomes in the two groups was depicted in Table 2. 

**Table 2:** A summary of outcomes between the two groups

**Variable**	**Meek**	**Mesh**	*p* ** value**
TBSA	44%	42%	>0.05
graft rejection	3 (15%)	5 (25%)	>0.05
Mean time of re-epithelization, Mean±SD, months	2.8±2.5	5.0±2.1	0.01
Average duration of operation, minutes	26.9±5.4	32.1±7.4	0.04
Expansion ratio	5.7±1.9	4.2±1.8	0.04
Local wound infection	3 (15%)	5 (25%)	>0.05

TBSA: Total body surface area

## DISCUSSION

Treatments in patients with extensive burns include maintaining homeostasis, nitrogen balance, enhancing immunity and preventing infection. The faster the patient’s wounds heal, the sooner the patient’s general condition is favored. Patients with large burn surface areas have been always a challenge for covering. A variety of techniques and materials either synthetic or skin processed products have been introduced to cover the wound. These include biological dressings, amniotic membranes, as well as synthetic dressings. All of which have their own disadvantages.^17^^-^^19^ Patient own skin, if available, is the best option. Therefore, Meek technique has been introduced to provide a higher expansion rate. 

 Seven children with burns ranging from 30% to 70% were assessed using Meek technique. Mean hospital stay was 51 days with an average of 3.3 procedures. Less than 3% of grafted areas needed re-grafting. The study showed that Meek had a high expansion ratio and could cover more areas and appeared to be effective in the management of patients with high surface area burns.^20^ Two patients developed wound infections which was slightly higher than our study. Duration of hospital stay was also more. 

In China, Meek and mesh techniques were compared in two groups each including 12 patients. Meek technique was shown to have lower therapeutic cost and better therapeutic effects compared to mesh.^21^ We did not compare costs because we performed both techniques in different limbs/areas of the same patients. However, the re-epithelialization rate in the Meek technique was lower in our study as well. Moreover, a large clinical trial was undertaken on 37 patients over a 5-year period. The mean TBSA was 72.9% and the mean grade III burn was 41%. The mean number of required surgeries was 1.84 and the survival rate after 5 years was 92%. The Meek technique could cover large skin burn wounds with good long term outcomes.^15^ However, in this cross-sectional study, two modalities like ours were not compared. 

In 2007, the Meek operation was assessed in 10 patients and compared with Stamp-like grafts (5 pts), micro-grafts (4 pts) or net-like grafts (1 pts) in other body areas of the same body. The Meek technique took less time to be completed and also with higher survival rate.^22^ We could not assess the fulltext as it was in Chinese, but the question remains how the survival rates were reported to be higher, when the control arm was another region in the same individual. However, only one patient in the control arm underwent net-like graft (mesh technique), which did not allow a reliable judgment and comparison. Despite this fact, the time of operation was less, which was in line with our finding. 

In 1994, expansion ratio using the Meek technique increased to 1;9, and the Meek technique was shown to be a practical substitute for mesh grafts, especially when there is paucity of donor site in larger surface burn wounds.^14^ This is in line with our study, as we showed that expansion ratio was higher in modified Meek technique compared to mesh procedure with a statistically significant difference. In 2001, the efficacy and high expansion rate of Meek technique were illustrated in seven patients suffering from severe burns,^13^ a comparative design was lacking.^14^

In 2016, the Meek operation was used in ten patients with a mean of 68±9.2% TBSA. Wound infection in all patients was visible; however, the rate of re-graft was 13.1±6.4% TBSA.^23^ In our study, local wound infection occurred only in 3 cases of the Meek group, which is significantly lower. However, the authors of this trial claimed that they were using the Meek technique routinely in their institution.^23^ Graft failure was also more than our study. However, their courage to use it routinely in their institute was admirable. 

As mentioned above, many clinical trials claimed the efficacy and superiority of the Meek technique over other techniques. However, there is paucity of case-control trials comparing the Meek technique with mesh, especially at different body areas of the same individuals. The strengthen points of our study was relatively larger sample size compared to other similar investigations and also its case-control design to perform the two techniques in different limbs/areas of the same patients. Performing the two techniques concurrently in the same individuals removed some bias regarding host factors and allowed for a more conclusive assessment. 

The limitations of our study were disability to compare patients’ survival rate or expenses between the two groups, which was not possible due to our design. Also, we excluded patients with diabetes. It is recommended to perform multicentric larger sample size case-control studies and include patients with underlying diseases like diabetes mellitus to fully elucidate all aspects of the Meek technique. Also, it is recommended for scientific burn societies to have a special look on the results of this technique in different trials and prepare preliminary guidelines to suggest it as a routine procedure for high surface burn wounds.

## CONCLUSION

 Mean time of re-epithelialization was lower in the Meek group with a statistically significant difference. However, the average duration of operation in modified Meek technique was lower and patients’ satisfaction was more. The expansion ratio was also higher with a statistically significant difference. Therefore, it is recommended to consider the Meek technique as a routine procedure, especially in those with high surface area burns. 
